# Feeding in the Digital Age: An Observational Analysis of Mobile Device Use during Family Meals at Fast Food Restaurants in Italy

**DOI:** 10.3390/ijerph17176077

**Published:** 2020-08-21

**Authors:** Allison Kiefner-Burmeister, Sarah Domoff, Jenny Radesky

**Affiliations:** 1 Department of Psychology, The University of Findlay, Findlay, OH 45840, USA; 2 Department of Psychology, Central Michigan University, Mount Pleasant, MI 48859, USA; domof1se@cmich.edu; 3 Department of Pediatrics, The University of Michigan, Ann Arbor, MI 48109, USA; jradesky@med.umich.edu

**Keywords:** parental internet use, parental device use, phone use, family mealtime

## Abstract

Caregiver mobile phone use while monitoring children is a behavior of increasing prevalence. Family mealtimes have long been considered a time in which parents and children connect emotionally and model eating behaviors, but prior studies have documented less parent-child conversation and more negative parent reactions to child behavioral bids at the table during parent phone use. Research on this topic to date is sparse and focuses only on US populations. The current study used non-participant naturalistic observation to record data on parental mealtime device use and family interactions while dining with children in a fast food restaurant in Italy. Thirty seven families (individuals observed = 129) were observed at a restaurant for the duration of their meal. Qualitative analysis of field notes indicated that mobile phones are frequently used by caregivers during feeding interactions. A novel observation in the current study was different patterns of use by parent gender and age. Mothers appeared to divide their attention between phone and child, whereas fathers evidenced more continuous mobile phone use during which there was lower responsiveness towards children during the meal. Younger parents more frequently used mobile phones, compared to older parents. Parents who missed child bids for attention were all phone users during the mealtime.

## 1. Introduction

Familial communication and interactions during mealtimes are greatly influential to the developing child with regard to both physiological and psychological health. Eating together as a family relates positively to the health of the child and parent [1]. Parental monitoring of child eating and modeling of healthy food related behaviors during mealtimes are of vital importance to the child’s future food related behaviors [2]. When caregivers are preoccupied during mealtimes, they may not be mindfully instructing their children on healthy habits or engaging in healthy feeding behaviors, such as modeling healthy eating. While parental distraction may lessen harmful feeding practices, like coercion, it also lessens opportunities for health education. One form of distraction is parental engagement with electronics. The use of electronic hand held devices during family mealtimes is both a ubiquitous problem and an understudied construct [3]. In addition to increasing unhealthy family eating and child obesity, the use of electronics such as television and mobile devices during family mealtimes may impede family bonding and interconnectedness [3,4].

One challenge of the permeating nature of mobile technology is its presence in physical and social spaces that may lead to user distraction [5]. Calling, texting, and web browsing during daily activities may contribute to physical harm or social relationship difficulties [5,6,7,8]. Mobile device use during social events has been associated with less communication among family members [9]. Parent and other caregiver mobile device use during interactions with children is common [10], yet to this point, relatively understudied [11]. Researchers have noted that while caregivers may be present physically, they are not attending to their child when they are highly absorbed in their mobile devices [3]. Indeed, absorbed device use by caregivers has been observed to co-occur with lower rates of parent-child interaction, missed bids for child attention, and harsher parent reactions to child misbehavior [3]. One important arena of parent-child interaction in which phone use may be particularly salient is the family mealtime [3,12].

While caregiver use of mobile phones during family mealtimes remains largely under-investigated, one study using nonparticipant observations in fast food restaurants in the US documented high rates of caregiver mealtime media use in July and August of 2013 (>70%; [3]). In this study, higher parent absorption with devices often co-occurred with lower parent responsivity, conversation, and sensitivity. Similar results were found by Hiniker and colleagues [11] while observing caregiving behaviors in Seattle area playgrounds. In these observations, caregivers using devices showed slower responsiveness to children’s bids for attention. Some reasons for caregiver device use have been provided by lab setting observations and interviews [13,14]. In a mealtime lab setting observation, device using parents showed less interaction with their children than non-device using parents and showed a tendency toward using their devices more in times of greater stress [13]. While sensitive subjects, such as improper parenting behaviors, are frequently studied through observation, self-report research of mealtime parental technological distraction has also shown relationships with child health behaviors such as lowered neophobia and heightened eating in the absence of hunger [15]. In interviews, parents of young children reported that device use was a method of stress alleviation and emotion regulation [14]. In addition to child differences, is known that parents of different genders tend to feed differently and assume different roles while feeding, therefore gender is also an important component of research in this area [16]. It has not been examined whether similar patterns of phone use occur in international populations, whether phone use differs based on parent demographics, or whether patterns of behavior are changing as smartphone ownership becomes more common [17].

To address this gap in the literature, the current study sought to examine caregiver behaviors during mobile phone use outside of the USA and explore whether phone use behaviors vary by parent gender or age. Naturalistic observation was used to investigate these parenting behaviors in a fast food restaurant in Italy. This methodology allowed for the examination of real-time behaviors as they would naturally occur. The institutional review board of the University of Dayton declared this study exempt from review.

## 2. Materials and Methods

### 2.1. Field Note Observations Procedure

Using naturalistic, anonymous nonparticipant observation, 37 family meals were studied at a fast food restaurant in a large Italian city (*N* individuals observed = 129, see Table 1 for demographic information). The restaurant was chosen for its “family friendly” nature and its proximity to a metropolitan transit station, which afforded a large and diverse study population. Observations were performed on weekend days and weekdays in May and June. Observations were made on eight separate weekdays between 10 am and 2:30 pm, with most observations falling between the 12 pm to 1 pm lunch hour as this was the best opportunity to view families eating within the normative Italian meal schedule.

All observations were performed by a developmental psychologist specializing in child and adolescent health behaviors. Field notes were taken with pen and paper in a notebook. Most observations were made from a high-top counter in the middle of the restaurant, enabling clear observation of families within 20 feet of the examiner. During almost all observation days, the restaurant had either reached or was near seating capacity (roughly 300 patrons).

Families included any adult with a child (estimated age was pre-pubescent/0–11 years old) whose mealtime could be closely observed from beginning to end. Child and caregiver age was estimated based on height, body structure/proportion, and overall developmental status (Table 1). Following the methodology and field note structure used by [3] family interaction during mealtimes were recorded via detailed notes regarding individuals’ demographic information (including number of family members, ages, genders, and inferred relationship), eating/feeding behaviors, affect, communication behaviors, and mobile phone use.

Families to be observed were generally identified while in line to order food or while walking to a table. The crowded, large fast food restaurant was generally populated by adult groups without children. Roughly 10–20% of customer groups had a child in their family as determined from estimation performed on three random days of data collection. Families with children were therefore noticeable to the researcher and were identified immediately once the researcher had completed the last observation. The highest number of children and caregivers observed in one group was four. While no school groups were apparent, it is nevertheless possible that an unrelated group was determined to be a family. All observable groups with children were included with no exclusions made. Observations began once the family meal commenced and detailed notes were taken for at least ten minutes or until meal was completed. Start and stop times of the meal were recorded in the field notes. The average length of meals was 16 min, with two meals lasting under ten minutes (seven and nine minutes, respectively).

### 2.2. Thematic Analysis

All field notes were transcribed, read, and coded by three cross-disciplinary experts (i.e., developmental-behavioral pediatrics, child clinical psychology, developmental psychology). Themes of media use and parenting behaviors were extracted from the reviewed transcripts and analyzed. The current study employed a grounded theory approach [18] to identify themes of parental phone use, parent behavior, and child behavior. This approach allows for the review of field notes to generate themes of investigation. This pattern of theme generation lessens the biases that result from using preconceived themes. The qualitative data review from grounded theory allowed novel themes to develop in addition to those investigated by Radesky and colleagues [3]. Each of the three investigators independently reviewed the field notes from the 37 observations. The most central and salient themes that emerged were discussed and agreed upon among the reviewers. In addition to the qualitative analysis, aspects of the phone use and parent-child interaction were also coded based upon thematic analysis. Codes for phone use and parent-child interaction were generated and applied to each field note transcript. In addition, field notes were coded for family demographics. Although the actual familial structure of the observed families may have differed (e.g., nannies, older siblings, mother and her partner), in the effort of clarity all caregivers outwardly identifying as men are hereafter referred to as father and those outwardly identifying as women are hereafter referred to as mother unless specific circumstances noted in the observation strongly implied a different relationship (e.g., grandparent).

### 2.3. Coding

Field notes were coded to quantify the following three observed behaviors: parent mobile phone use, child bid for attention, and missed bid for attention (defined below). A subset of field notes was double coded and reliability was checked. Coders had acceptable reliability for each of the following codes (κ’s > 0.80). Although frequency of these codes are demonstrated in the Results section, we did not perform bivariate statistical analysis due to the small sample size, and the frequencies are displayed for descriptive purposes.

### 2.4. Parent Mobile Phone Use

Parents’ mobile phone use was coded as (0) no use; (1) brief/intermittent use or presence only on the table; or (2) frequent/continuous. If two parents were present, both parents’ mobile phone use were coded separately (κ = 0.81 and 0.92). Parents were coded as intermittent users if they used their phone briefly (duration) and rarely (i.e., once or twice throughout the meal for a few seconds only). For example, parents who ‘checked’ their phones were coded as having brief/intermittent phone use. Parents were coded as having frequent or continuous phone use if they were on their phone for the majority of the meal or if they did not divert eye gaze from the mobile phone for long periods of time (i.e., they were absorbed).

### 2.5. Child Bid for Attention

Field notes were coded to identify whether or not the child *ever* requested the parent’s attention (κ = 0.86). Children were coded as having requested attention from parents (i.e., bid for attention) if they reached out for a parent either physically or verbally. In order for a bid for attention to take place, the parent needed to have been focusing their gaze and their physical focus (e.g., hand placement) elsewhere. When the parent was engaged in phone use, examples of bids included the child attempting to avert their gaze from behind the phone or pull the phone away from the parent’s face. When the parent was not engaged in phone use, examples of bids included a hand wave or verbal call.

### 2.6. Missed Bid for Attention

A bid for attention was coded as “missed” if, at all during the meal, a parent missed or did not attend to the child’s attention bid (κ = 0.86). Specifically, if the parent did not attend visually, attend physically, or verbally respond to the child after an attention bid and before the child began to occupy themselves once again, the field note was coded as having a missed bid for attention.

## 3. Results

### 3.1. Sample Characteristics

Thirty-seven families were observed eating a meal at a fast food restaurant in the historic area of downtown Florence, Italy in May and June 2016. The restaurant was patronized by a heterogeneous international clientele due to its close proximity to a major metropolitan train station. Observed families were diverse in terms of estimated caregiver and child age and language spoken (Table 1). The majority of families observed had one child (56.8%) and two caregivers (56.8%). The number of caregivers and the number of children both ranged from 1 to 4. Twenty-two of the 37 families were observed to be in possession of a mobile phone during the meal (59.5%).

#### 3.1.1. Theme 1: Gender Differences in Phone Use

Men and women appeared to differ both on how much they used their phones and how absorbed they were in their phone use while monitoring their children (See Table 1 and Table 2). Male phone users often appeared to be continuous users and absorbed into their phones while in use. Fathers who used their phones continuously did not appear to attend to their child’s behaviors. While this pattern was more apparent when a second caregiver was present to share responsibility (in the observed cases the second caregiver was always female), even fathers who were alone with a child showed a low level of parent-child synchrony and communication.

This example of a continuous use father showed how child attention shifted when the father was alone and then with his partner.

“Father is on his phone and the baby is quiet [about 7 months old]. He glances at the baby, then back to his phone scrolling [maybe on internet]. Not typing. He gets baby’s attention by tapping his hand on the table in front of her to the beat of the radio [while looking at phone]. He looks at her and tickle/pinches her, then goes back to his phone. He is very engaged with his phone. He engages with baby for 1–2 s every 30 s or so and then is back to his phone which was either on table or being held about a foot from his face. Mother returns. He shows her photos on his phone and they laugh. Adults talk bored/content. She is engaging with baby. He is eating while on his phone. She drinks coffee and talks to the baby. She leans toward baby. She feeds the baby with a bottle. He watches, no longer on his phone, then he is back on his phone while she feeds the baby milk from a bottle. He finishes his burger while poking at his phone on the table not engaging with her or the baby. She and the baby engage with one another. Adults speak inaudibly. Baby is eating and content. He is silent and on his phone while his face is flat/neutral in expression most of the time. She smiles and reacts to the baby quickly. Baby holds [mother’s] hands while being fed… Baby smacks table for attention playfully. Mother immediately ignores phone and engages baby using hand play. Father never looks up from phone. Mother takes baby out of chair and bounces her on lap, kisses her playfully. Father never looks up from phone or speaks.”

Mothers who were coded as continuous users tended to split their attention between their children and phone.

“Father is still scrolling on his phone. He is silent and nearly motionless. Mother wipes hair from girl’s face [3–4 years old]. Adults are waiting for the girl to finish. Girl eats slowly but steadily while bouncing. Then the mother was on her phone. Father looks around and out the window. Mother scrolls on her phone seeming to be on the internet. Adults are not on phones at the same time. Girl watches mother on the phone. Mother’s face is always pleasant/neutral. Girl clings to mother’s arm and watches her on her phone. Girl then begins to fling herself around the booth. Father finishes the girl’s milkshake. Mother shows the girl her phone again... Father is also on his phone. Always scrolling/internet. The girl then gets under the table. Then she eats her burger and bounces.”

In this second example of a continuous using mother, the mother of two young school age children [about 4 and 5 years old] shows a systematic pattern of split attention between children and phone.

“This pattern was a system of attending to the child and the phone as a loop. She would scroll on her phone, then look at the kids, then look around the room, then back to the phone. When her children required attention, they were immediately acknowledged. However, little to no conversation or interaction was seen while the children were eating.”

#### 3.1.2. Theme 2: Child Bids for Attention

Many families had children who displayed behavioral bids for attention. Babies and young toddlers displayed mostly calling and reaching behaviors, while preschoolers and school age children generally solicited attention by way of boisterous or goofy actions. For example, one preschool girl attempted to gain the focus of her father and/or mother by singing, crawling under the table, and even by playfully falling off of her crouched feet onto the booth seat. Her father was a continuous and highly absorbed phone user, while her mother was a continuous user, but frequently looked up or toward her child and only used her phone for scrolling purposes toward the end of the meal.

In this sample, the small subsample of families who did miss a child bid for attention were all phone-using caregivers—sometimes missing bids while on the mobile phone, and other times not (see Table 3).

One intermittent phone using mother was texting while her preschooler [about 3 years old] grabbed for her attention by drinking her beer. This attention grab was successful, as the mother removed the beer can. Other attention grabs from this child were ignored by the mother while she was not on her phone including another drink of beer and an outburst of shouting and wall pounding.

“Boy tries to get under the table and is lightly scolded. Mother is texting and while mother is distracted texting with the phone about a foot from her face the boy sips from the lid of the beer can. Mother looks up from her text and snatches the can away. She does not seem angry; she is just moving the can. The boy holds his burger and nibbles on it but he is more interested in exploring his arms, his body, and the table. Mother holds the phone up a foot from her face and texts with her pinky… Boy is standing on the seat shouting and pounding on the wall. Mother shows almost no reaction to his behavior. Now the boy is seated and wearing trash like a hat.”

Another example of missing attention bids was shown in one father who was the sole caregiver for a female preschooler.

“The child was in the presence of her caregiver, but was left unviewed for over 5 min while sitting across the table from him. She pointed things at him, stole fries from him in, stacked all of the trash into a tower on his side of the tray, messed with his items, and finally climbed over the table at him. It was only after this last attention grab that he talked to her while still scrolling on his phone. She then gets onto the floor by him and receives a side hug as he scrolls on his phone with the other hand.”

#### 3.1.3. Theme 3: Age Differences in Parental Phone Use

Younger parents (under 40 years old) were more often on their phones while with their children than middle-aged parents (40–59 years old). Even when caregiving for younger children, middle-aged adults generally stayed off of their phones completely.

Older adults (60+ years old) were both infrequently observed to be with young children, and unlikely to use phones when they were with young children. The only older adult to use a mobile phone was one older adult man who took one photo of the family.

Parents of very young children (under school age) appeared to be the heaviest phone users during observations followed by parents of school age children. Many observed families had one younger child along with one or more teenagers (see Table 1). Teens were not often observed using phones in this sample (with one exception). Further, parents with teenagers in this sample were more likely to engage in consistent conversation with their teens than parents with younger children and never engaged in phone use themselves.

#### 3.1.4. Theme 4: Boredom

Observed families displayed numerous apparent reasons for mobile phone use. Many of these motivations were clear to the researcher as purposeful (e.g., maps), while other reasons were unclear (e.g., texting or scrolling). Generally, it seemed that parents engaged in more purposeful uses due to opportunity and regardless of observed emotion. Beyond these purposeful phone uses, however, many parents engaged in phone use after showing outward signs of boredom (e.g., facial boredom, searching the environment for stimulation). Frequently, parents finished eating before their children and appeared to be searching for something to entertain them while their children were eating.

One continuous phone using mother of two young children [about 4–5 years old] was observed searching for something to do on her phone and sighed and momentarily put the phone down repeatedly (the researcher assumed this was because nothing entertaining was found).

“Mother is scrolling on her cell phone. Phone is held at first—now on table. Now she holds it about 6 inches from her face and scrolls like she is reading or on the internet. Now she takes photos of the kids. And puts the phone on the table. She watches the kids eat. She holds the phone and talks to the girl. Boy watches other people all around him. Mother is back on the phone in front of her face. Mother is scrolling on the phone with a bored expression. Mother always doing rounds with her gaze—kids, phone, around room, kids, phone, around room.”

In this next example, the mother attempts to engage with the child [boy about 6 years old] before showing outward signs of boredom, eating food scraps, and reading an article on her phone while waiting.

“Mother seems to be finished with her food. Mother is very patient and waits for the boy to eat. Mother seems bored and eats fries slowly (this fry eating seems to be only for entertainment). Boy hands over his toy and is finishing his burger. Boy is still eating and mother is finishing her drink they talk and mother tries to get response but boy ignores her and eats while staring out the window. Mother eat a piece of boy’s burger. Mother is on her phone and reading an article. She is attending to the boy while reading with speaking and glancing. Until now mother has not used the phone except check charge.”

## 4. Discussion

The purpose of this study was to examine caregiver mobile phone use and parenting behaviors during mealtimes in fast food restaurants in an international setting, as prior studies have only examined US populations. Our observational study revealed that, similar to what has been found during US fast food meals [3], mobile devices are frequently used by caregivers during feeding interactions. A novel observation in our study was different patterns of use by parent gender and age. Mothers appeared to divide their attention between their phone and child, whereas fathers evidenced more continuous mobile phone use during which there was lower responsiveness towards children. Younger parents frequently used mobile phones, compared to older parents. Finally, also consistent with prior research [3], parents who missed child bids for attention were phone users during the mealtime. Taken together, our study supports prior findings of parental phone use during family meals in fast food restaurants, and offers preliminary hypothesis-generating evidence for demographic (gender, age) differences in parental phone use during mealtime.

As females are often the caregivers responsible for feeding [19], it may not be surprising that gender differences emerged in our observations. However, recent research also suggests that there could be other reasons for these gender differences. McDaniel and Radesky [20] discovered that technological distractions during mother-child interactions were associated with greater internalizing and externalizing symptoms in children, whereas such distractions did not associate with these behaviors in father-child interactions. As McDaniel and Radesky [20] note, children behave and regulate their affect differently when in the presence of fathers compared to mothers [21]. Thus, children may expect differential responses during parent-child interactions (i.e., they may expect mothers to be more responsive), possibly accounting for the gender differences in caregiver phone use during family interactions.

Age differences are also not surprising given that younger adults have higher rates of smart phone ownership [17]. Although this is based on a small sample and thus not powered or designed to test for significance, future quantitative research should confirm these factors (gender, age) and examine other factors that may influence parent mobile phone use during feeding, as this could be influential for the mealtime environment. For example, boredom appeared to contribute to parental phone use (e.g., while waiting for the child to finish eating), which has been documented in prior studies of adult mobile device use [22]. Further, parents in focus group interviews similarly reported using digital media to withdraw from social interactions, and in response to parenting stress and boredom [14]. As such, motivations for using mobile phones during contexts that could be child-centered (and contexts wherein parent-child interactions are health-promoting) should be examined, as this may present barriers to changing mobile phone use by parents.

Mobile device use co-occurred with child bids for attention and missed bids for attention. Certainly, this type of technological distraction may limit the frequency of positive and responsive parent-child interactions (as was found in a study in the US, [3]). The protective effects of family mealtimes on child obesity is suggested to be as a result of healthy family communication and interaction during the mealtime context [23]. As such, it is possible that the presence of mobile phones during mealtimes may weaken these protective factors. Longitudinal research should test this hypothesis as well as whether this type of tech use reduces health promoting interactions between caregivers and children during mealtimes.

There are a few limitations that should be acknowledged. The restaurant used for observations offered wireless internet, but only through a system of signing in, which may have deterred some parents from logging on (thus may contribute to lower mobile phone use than was observed in this sample). As a large number of observed families appeared to be travelling (evidenced by suitcases), it is possible that many did not have access to the internet during their visit. Accounts and reports of cell phone use in this study are, therefore, likely under representative of phone use in domestic situations. Also, though not the purpose of observational research, it is still important to state that our findings may not be generalizable to the entire Italian population. For an in-depth examination of Italian parent-child mealtime interactions see [24]. Thus, future studies should seek to replicate these findings in a larger, demographically representative sample. Further, using multiple restaurants and observers would have enhanced this study and should be considered in future studies. Lastly, this study generally relied on heteronormative gender roles while observing and reporting data. While recent research has supported the consistency with which parents propagate heteronormative feeding styles researchers should work to minimize their dependence on these structures [25]. The use of the terms ‘mother’ and ‘father’ throughout the manuscript was a stylistic choice for ease of reading, but potentially misidentified participants’ relationships and genders. Future research should work to further the dissolution of heteronormative reliance in social science research.

## 5. Conclusions

Mobile phone use is increasingly common during parent-child activities, and as users adapt to the challenges of balancing technology with interpersonal spaces/interactions, it is important to know how family behaviors are changing. Our observations support current clinical recommendations for unplugged or no screen time during family meals, particularly given the occurrence of missed child bids in families with parental mobile phone use. Although naturalistic observations remain an important methodological approach to examining mobile phone use during meals and other family interactions (given the limitations of self-report and lab-based studies, in which users might have difficulty recalling phone use and/or change their behaviors, respectively), longitudinal research is needed to examine associations between parental mobile phone use during meal time and child obesity risk in Italy and other European nations.

## Figures and Tables

**Table 1:** Family demographic and phone use information.

	*n* (%)	Any Parent	Any Parent	Continuous Use
		Phone Use	*n* (%)	*n* (%)
		*n* (%)		
**Caregiver Gender**				
Female	41 (61%)	13 (32%)	10 (77%)	3 (23%)
Male	26 (39%)	11 (42%)	6 (55%)	5 (45%)
**Caregiver Age**				
**Categories**				
20–39	31 (46%)	18 (58%)	12 (67%)	6 (33%)
40–59	32 (48%)	5 (16%)	3 (60%)	2 (40%)
60+	4 (6%)	1 (25%)	1 (100%)	0 (0%)
**Family Language**				
Italian	11 (30%)	5 (46%)		
Other European Origin	8 (22%)	6 (63%)		
Asian Origin	2 (5%)	1 (50%)		
Inaudible/Unidentifiable	16 (43%)	6 (50%)		
**Child Gender**				
Female	34 (54%)	17 (50%)		
Male	29 (46%)	12 (41%)		
**Child Age Categories**				
Infant (Under 1 year)	4 (50%)	3 (75%)		
Toddler (1–3 years)	2 (23%)	2 (100%)		
Preschool (3–5 years)	9 (50%)	8 (89%)		
School Age (5–11 years)	40 (23%)	16 (33%)		
Teenage (12–18 years)	7 (50%)	0 (0%)		

Note: Families with at least one child 0–11 years were observed. In the instance that teenage siblings accompanied school age children, the teens were observed as well.

**Table 2:** Type of parental phone use by youngest child in family.

	Infant	Toddler	Preschooler	School Age
**Father**				
Brief/Intermittent	0	0	2	4
Frequent/Continuous	2	0	3	0
**Mother**				
Brief/Intermittent	2	2	2	4
Frequent/Continuous	0	0	3	0

Note: While 43% of observed families had multiple children, this table examines parental phone use type by only the youngest child in each family. All phone using adults are included in this table, but only the youngest or only child in each family is represented.

**Table 3:** Child bids for attention.

	Phone Using Parent	Non-Phone Using Parent
Child Bid for Attention	10 (45%)	1 (7%)
Missed Bid for Attention	5 (50%)	0 (0%)

Note: Both intermittent and continuous phone-using parents are categorized as.phone using parents in this table. Percentage of child bids for attention denotes percentage of families in phone using category (user or non-user) who had a child bid for attention at least once. Percentage of missed bid for attention denotes the percentage of missed bids for attention in each phone using category.
